# Developing a Health Literacy Scale for adults in Hong Kong: A modified e‐Delphi study with healthcare consumers and providers

**DOI:** 10.1111/hex.13651

**Published:** 2022-11-08

**Authors:** Cindy Yue Tian, Eliza Lai‐Yi Wong, Richard H. Xu, Annie Wai‐Ling Cheung, Dong Dong, Phoenix K.‐H. Mo

**Affiliations:** ^1^ JC School of Public Health and Primary Care, Faculty of Medicine The Chinese University of Hong Kong Hong Kong China; ^2^ Center for Health Systems and Policy Research, JC School of Public Health and Primary Care, Centre for Health Systems and Policy Research The Chinese University of Hong Kong Hong Kong China; ^3^ Department of Rehabilitation Sciences The Hong Kong Polytechnic University Hong Kong China

**Keywords:** Delphi survey, Health Literacy Scale, Hong Kong Chinese, mixed methods research

## Abstract

**Introduction:**

Health literacy (HL) refers to individuals' abilities to process and use health information to promote health. This study aimed to develop the first HL measurement tool for the Chinese Hong Kong population.

**Methods:**

A two‐phase methodology was adopted. In *Phase I*, evidence synthesis with a deductive method was conducted to formulate the item list from the literature. In *Phase II*, a modified e‐Delphi survey was conducted among stakeholders (i.e., healthcare providers and healthcare consumers) to confirm the content validity of the item list. The stakeholders were invited to rate the relevance of each draft item on a 4‐point scale and provide suggestions for revisions, removal or adding new items.

**Results:**

In *Phase I*, a total of 34 items covering functional, interactive and critical HL were generated. In *Phase II*, to obtain a balanced view from experts and laypeople, healthcare professionals (*n* = 12) and consumers (*n* = 12) were invited to participate in the Delphi panel. The response rates of the three rounds were 100%. After the third round, the consensus was reached for 31 items, and no further comments for adding or revising items were received. All items exhibited excellent content validity (item content validity index: 0.79–1.00; K*: 0.74–1.00).

**Conclusions:**

A Health Literacy Scale for Hong Kong was developed. Compared with existing HL scales, the scale fully operationalized the skills involved in functional, interactive and critical HL. The Delphi study shows evidence supporting the high content validity of all items in the scale. In future studies, these items should undergo rigorous testing to examine their psychometric properties in our target population groups. By illuminating the details in the development process, this paper provides a deeper understanding of the scale's scope and limitations for others who are interested in using this tool.

**Patient or Public Contribution:**

Public as healthcare consumers, in addition to healthcare providers, were involved in developing a new HL scale for this study. The input from the public contributed to examining the scale's content validity by judging whether all items reflected the skills that they need to find and use health‐related information in their daily life.

## 1 INTRODUCTION

Health literacy (HL) is defined as an individual's capacity to obtain and process health information to promote health.^1^ It can contribute to how people interpret symptoms and participate in health‐related decision‐making. Limited HL has consistently been associated with poorer self‐reported health,^2^, ^3^ lower health‐related quality of life,^4^ less use of preventive health services,^5^ increased hospitalizations^6^ and higher healthcare costs.^7^, ^8^ Many national surveys have highlighted high rates of poor HL in populations.^9^, ^10^, ^11^, ^12^ Previous systematic reviews indicated that the prevalence of low HL in Europe ranged from 27% to 48%,^13^ while in Southeast Asia it ranged from 1.6% to 99.5% with a mean of 55.3%,^14^ depending on the literacy measurement method applied. The most common factors associated with insufficient HL include educational attainment, age, income and ethnicity.^13^, ^14^

Identifying a relevant measurement is critical for examining HL levels. Early efforts to measure HL primarily focussed on individuals' abilities to read and comprehend health‐related materials in a clinical setting.^15^, ^16^ With healthcare shifting from a clinical setting to a community setting, more recently developed measurement tools measure a broader understanding of HL, which includes a set of competencies (e.g., information‐seeking skills, communication skills and decision‐making skills) needed to facilitate health decision‐making in both clinical and nonclinical settings.^17^, ^18^, ^19^, ^20^ Although over 100 HL scales (HLSs) have been developed, no widely adopted measurement tool could reflect our current understanding of HL.^21^, ^22^, ^23^ Taking the most cited HL tools as examples, the Test of Functional Health Literacy,^15^ Rapid Estimate of Adult Literacy in Medicine and Newest Vital Sign^16^ narrowly measure basic skills and knowledge of health; The Health Literacy Questionnaire^17^ did not include the ability to address the broader goal of promoting health and reducing health disparities among individuals and communities. Moreover, most available HL tools were developed in Western countries.^24^ Hence, discussion about HL scale development in Asia is still needed.

Scholars have argued that a robust HL scale should allow for discovering new knowledge and testing what we know from previous studies to advance this field.^23^, ^25^, ^26^ Therefore, using a testable theory to support the creation of a new scale is vital. The present study is based on Nutbeam's framework of HL, which is widely used in this research area. This framework divides the main skills associated with HL into three levels: functional health literacy (FHL) referring to individuals' basic literacy and numeracy skills (e.g., being able to read and write, basic knowledge of health) to access and act upon health‐related materials; interactive health literacy (IHL) referring to individuals' cognitive and social skills to extract information from all kinds of forms of communication and to interact with information providers for achieving better health outcomes (e.g., searching for online health information and requesting clarification during healthcare consulting) and critical health literacy (CHL), which refers to individuals' higher level cognitive and social skills which can be applied to critically analyse information, and to use this information to gain better control over life events that impact health, such as disease management and health promotion.^27^ Nutbeam's framework synthesizes HL skills in a comprehensive way compared to other frameworks used in HL research. For instance, the Chinese Resident Health Literacy Scale adopted ‘basic knowledge and skills of people's health’ as the underlying structure, which mainly covered the skills involved in FHL.^28^, ^29^ The European Health Literacy Survey Questionnaire used Sørensen et al.s'^19^ theoretical model of ‘the competencies needed in the information processing’. The authors of this European scale admitted that the scale could not thoroughly assess an individual's ability to use the information to promote health, which is addressed in CHL.^19^

However, compared with FHL and IHL, CHL is not fully operationalized in current HL scales. As of writing, six scales covering Nutbeam's framework^30^, ^31^, ^32^, ^33^, ^34^, ^35^ and one scale measuring the single domain CHL^36^ for adults have been published. These studies^30^, ^31^, ^32^, ^33^, ^34^, ^35^, ^36^ mainly emphasized the ability involved in critical appraisal of information as the component of CHL. This emphasis, however, was not explicitly linked to the theory of this domain. Nutbeam initially highlighted that CHL includes not only the ability to critically assess the quality of information but also a range of competencies to enable individuals to realize social and structural factors influencing health and take actions to address these factors for better health.^27^ Among the above scales, only the All Aspect of Health Literacy Scale^30^ made efforts to examine the missing components of CHL: namely, knowledge of and actions to address social determinants of health. But the author admitted that there exist challenges to address this shortage. As such they only adopted three items involved in the capabilities for community empowerment and social engagement for health to indirectly reflect these understandings and actions.^30^ The above revealed that continuous discussion on effectively measuring this domain among adults is still needed.

In addition, there is no rigorously validated HL scale for the general population in Hong Kong. Although several studies explored the HL levels in Hong Kong, the scales they used were either condition‐specific (i.e., disease‐specific and population‐specific)^37^, ^38^, ^39^, ^40^, ^41^ or directly translated from existing scales without psychometric testing.^42^, ^43^ Hong Kong has a dual‐track healthcare system encompassing the public and private sectors. The downsides of the two sectors are the long waiting times experienced in public hospitals and high healthcare costs in private hospitals.^44^, ^45^ Under such circumstances, patients are expected to actively engage in self‐management, which requires a high HL level. It is reasonable to assume that patients with sufficient HL skills are more likely to understand their symptoms and be able to decide when and what healthcare service to utilize in the health system. Therefore, one reliable and valid HL scale is essential to understand residents' HL levels and design research‐based strategies to enhance HL in the local health system.

From all these perspectives, we aimed to develop a validated theoretical‐based HL scale (HLS‐HK) by adopting Nutbeam's framework^21^, ^22^, ^23^, ^24^, ^25^, ^26^ in Hong Kong. Previous studies mainly invited healthcare professionals to design HL scales.^16^, ^33^, ^46^, ^47^, ^48^ Considering HL is a critical component of people‐centred health care, which demands participation from the healthcare provider and consumer side,^49^, ^50^ we included healthcare providers and consumers in the scale development process. The purpose of this paper is therefore to highlight the development process and the content validity of the HLS‐HK via a modified e‐Delphi technique.

## 2 METHODS

The Delphi technique is a systematic and interactive method to achieve a general agreement or convergence of opinions on a particular topic.^51^ It has proven to be a reliable method to develop new concepts^52^ and establish consensus across a range of subject areas,^53^ including several in the field of HL measurements.^19^, ^54^, ^55^, ^56^ In the present study, two phases were conducted: (a) item development of HLS‐HK by evidence synthesis using a deductive method and (b) content validity of HLS‐HK employing a modified e‐Delphi survey with healthcare consumers and providers.

### 2.1 Phase I: Item development

A deductive method^43^ was used to generate items based on our previous two scoping reviews.^57^, ^58^

#### 2.1.1 Theoretical framework

We conducted two scoping reviews^57^, ^58^ to ensure the scope and coverage of the scale with the adoption of Nutbeam's framework. The first scoping review synthesized how Nutbeam's framework was operationalized in current HL scales.^57^ Given that CHL is the least well‐developed domain in Nutbeam's model, we conducted another scoping review to understand the components that need to be measured in this domain. By doing so, the following three subdomains of CHL were identified: CHL‐1: ‘critical appraisal of information’ is an individual's ability to evaluate the quality of information; CHL‐2: ‘understanding of social determinants of health’ coveys individual's understanding of the relationship between how people experience social determinants and the impact of these determinants on health; CHL‐3: ‘actions to address social determinants of health’ focusses on individual's competency to translate knowledge into actions to address the modifiable determinants of health.^58^ To sum up, a framework within five content areas (i.e., FHL, IHL and three subdomains of CHL) of this newly developed scale was developed.

#### 2.1.2 Item generation

Then, we turned these five abstract contents into measurable observations. A deductive analysis with the following three steps was performed to generate items: (1) sample: choosing reliable and validated scales with the indicators of interest from the two scoping reviews^57^, ^58^; (2) coding: labelling the content of identified items and then grouping the labels into content categories; (3) results: the final content categories served as the template for the generation of an item pool. The three‐step process was conducted by two researchers, and agreement was achieved through discussion with the research team. To ensure the coverage and minimize the cognitive burden, the number of items was expected to be between 30 and 50.

### 2.2 Phase II: Modified e‐Delphi study

A modified e‐Delphi survey was conducted to assess the content validity^59^ of items developed from Phase I.

#### 2.2.1 Participants

In Delphi exercises, 10–18 respondents are suggested as sufficient for ensuring consensus.^60^, ^61^, ^62^ We assembled a panel composed of healthcare providers (Group A) and healthcare consumers (Group B) via nonprobability purposive sampling. Regarding the inclusion criteria, according to Hasson et al.s'^63^ suggestion, participants in Group A were required to be healthcare professionals or clinical workers who had been working in the health field for ≥5 years. In Group B, participants were required to be permanent citizens aged ≥18 years and have experience in seeking health‐related information. Given that everyone should need healthcare information at some point, we proposed that every citizen could be a participant in Group B. To achieve a representative sample, we selected participants by considering a balance of different professional disciplines in Group A and a balance of gender, age and educational attainment in Group B. To keep the recruitment costs low, for Group A, we invited doctors and nurses from one public hospital and professors with experience in health‐related research from one local university. For Group B, we approached citizens who may be interested in joining our study, including staff and students in the local university and people who work outside the university. We expected at least three rounds of exercise to complete the Delphi process. Participants were required to take part in all three rounds. Therefore, if they did not respond to Round 2, they were not invited to participate in Round 3. This study aimed to recruit and complete the process with 20 participants and 10 respondents for each group.

#### 2.2.2 e‐Delphi rounds

We used Qualtrics software (version August 2021)^64^ to develop the online three‐round survey and invited potential participants via email or face‐to‐face. A 4‐point Likert‐type scale (ranging from 1 = not at all relevant to 4 = extremely relevant) was used to determine raters' agreement on item relevance. Ratings of 1 and 2 were considered ‘not relevant’, whereas ratings of 3 and 4 were considered ‘relevant’ as in most studies.^65^, ^66^ Additionally, text boxes were provided in the scale for raters to include comments and suggestions.

In Round 1, participants were asked to independently rate each drafted item for relevance on a 4‐point scale. They were also encouraged to add free‐text comments on the scale's design, clarity and content and suggest additional items that may be used to measure HL skills based on their knowledge and experience. Data on participants' demographics and expertise were also collected in this round. In Round 2, all participants received an individualized questionnaire that included all items from Round 1 which occurred alongside the participants' own responses and all participants' responses to each item. Participants were asked to reconsider their responses in light of the two groups' responses and item modification. Based on the comments we received from the previous round, we revised items and highlighted the changes in the questionnaire for rerating in this round. Additionally, the results on item relevance and a summary of comments of the previous round were provided to the panellists in Supporting Information: Appendix. In Round 3, each participant was asked to confirm the items after the previous round and reconsider their responses, considering the groups' responses for a final time. We also provided a summary of comments and highlighted the item modification from the previous round.

#### 2.2.3 Data analysis

Data analysis was carried out between each round using Microsoft Excel (version 16.63.1).^67^ Two approaches were used to calculate content validity. Item content validity index (I‐CVI) is the proportion of items that received a rating of 3 or 4 in terms of relevance by panellists. It can be calculated by using this formula: I‐CVI = *A*/*N* (*N* is the number of panellists; *A* is the number of panellists who agree it is relevant). It is recommended that if the I‐CVI > 0.79, the item is appropriate; if it is between 0.70 and 0.79, the item needs revision; and if the value is below 0.70, the content validity of the item is not acceptable and the item is eliminated.^65^, ^68^ Although I‐CVI is widely used to estimate content validity, the index does not consider the possibility of chance agreement. The second approach was the Kappa statistic (K*) which adjusts for chance agreement by examining interrater agreement. To calculate Kappa, the probability of chance agreement was first calculated for each item by the following formula: *p*_c_ = (*N*!/*A*! [*N* − *A*]!) × 0.5^*N*^. After calculating I‐CVI for all items, Kappa can be computed by using the following formula: K* =(I‐CVI − *p*_c_)/(1 − *p*_c_). Evaluation criteria for Kappa are as follows: if the values are larger than 0.74, between 0.70 and 0.74, and between 0.40 and 0.69 are considered as excellent, good and fair content validity, respectively.^69^ If the K* is equal to or above 0.70, the content validity of the item is acceptable. After each round, qualitative data were analysed and interpreted to clarify and confirm consensus around the wording.

**Figure 1:**
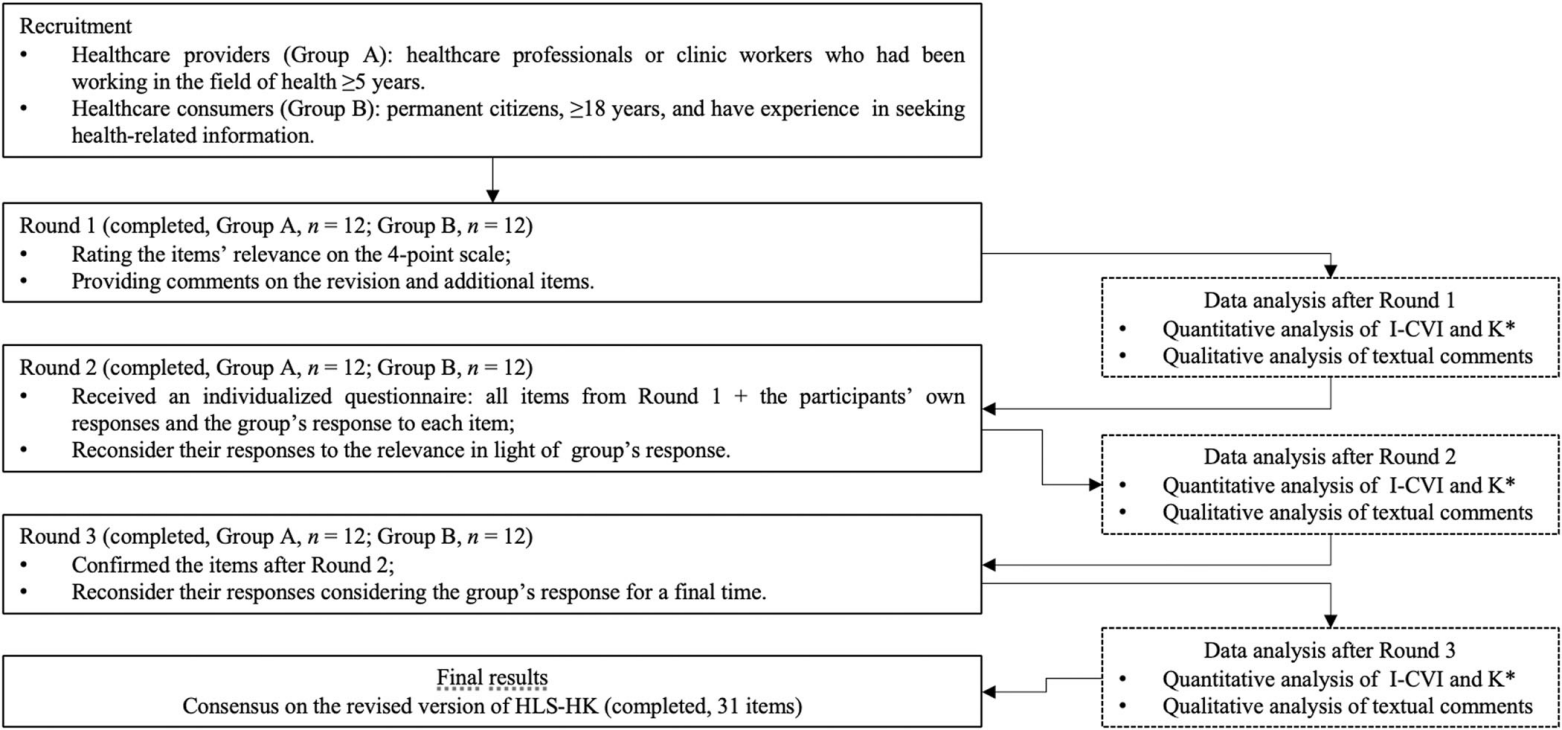
Summary of the Delphi process

A consensus was defined as ≥ 70% of all participants agreeing that one item is relevant in Round 3. We recruited the same number of participants in the two groups. We considered that all participants' responses were weighted equally, as with most studies.^70^, ^71^ In this way, the consensus could be achieved while avoiding the impact of dominant individuals and groups. Figure 1 provides a summary of the Delphi process.

## 3 RESULTS

### 3.1 Phase I

Using a three‐step deductive process, we identified seven tools^30^, ^31^, ^35^, ^48^, ^72^, ^73^, ^74^ and consolidated 34 items into the 5 relevant content categories (see Supporting Information: Appendix 1). Given that the items were originally formulated in English, a forward–backward translation was produced by four bilingual translators (two translators for each translation). After that, we performed one review meeting among the research team to determine the primary version of HLS‐HK in traditional Chinese.

### 3.2 Phase II

For the modified e‐Delphi Survey, a total of 24 experts from Group A and Group B participated in the survey from August to October 2021. All of them completed all three rounds of the survey with response rates of 100%. Table 1 presents the demographic characteristics of the respondents. The experts in Group A (*n* = 12) included six doctors, one nurse, two public health professors, two nursing professors and one social science professor. The participants in Group B (*n* = 12) covered two postdoctoral fellows, two university students and eight workers outside of academia. The three‐round survey indicated that the scale has good content validity (see Table 2). The consensus was reached for finalizing 31 items after three rounds (see Table 3). The wording changes and final Chinese version of the HLS‐HK can be found in Supporting Information: Appendices 2 and 3, respectively.

**Table 1:** Demographic characteristics of panellists

	Group A healthcare provider (*n* = 12)	Group B healthcare consumer (*n* = 12)	Total (*n* = 24)
Gender			
Male	9 (75.0%)	5 (42.0%）	14 (58.3%)
Female	3 (25.0%)	7 (58.0%）	10 (41.7%)
Age group			
18–29	0 (0.0%)	4 (33.3%）	4 (16.7%)
30–49	8 (66.7%)	5 (41.7%）	13 (54.2%)
≥ 50	4 (33.3%)	3 (25.0%）	7 (29.1%)
Education attainment			
Secondary or below	0 (0.0%)	2 (16.7%）	2 (8.3%)
Postsecondary (diploma/certificate course)	0 (0.0%)	1 (8.3%）	1 (4.2%)
Postsecondary (degree course)	12 (100.0%）	9 (75.0%）	21 (87.5%)
Diagnosed chronic disease			
Yes	5 (41.7%）	2 (16.7%）	7 (29.2%)
No	7 (58.3%）	10 (83.3%）	17 (70.8%)
Main work setting			
Academia	5 (41.7%)	2 (16.7%)	7 (29.2%)
Clinic	7 (58.3%)	0 (0.0%)	7 (29.2%)
Industry	0 (0.0%)	8 (67.7%)	8 (33.3%)
Others^a^	0 (0.0%)	2 (16.7%)	2 (8.3%)

^a^ Others refers to students.

**Table 2:** Content validity of items included in the scale (three‐round survey)

	I‐CVI	K*	No. of items					
			FHL	IHL	CHL‐1	CHL‐2	CHL‐3	Total
Round 1	0.75–0.96	0.74–1.00	5	7	7	9	6	34
Round 2	0.79–1.00	0.74–1.00	5	7	6	9	6	33
Round 3	0.79–1.00	0.79–1.00	5	7	6	7	6	31

Abbreviations: FHL, functional health literacy; I‐CVI, Item content validity index; IHL, interactive health literacy.

**Table 3:** Content validity of items included in the scale (Round 3)

Domain	No.	Item	I‐CVI	K*	Interpretation
FHL	1	*How often do you* ^a^: …need help when you are given information to read by your doctor, nurse or pharmacist	0.88	0.87	Excellent
	2	…need help when you are asked to fill out medical forms by your doctor, nurse or pharmacist	0.88	0.87	Excellent
	3	…find that characters cannot understand when you read instructions or leaflets from hospitals or clinics	0.79	0.79	Excellent
	4	…feel that the content is too difficult to understand when you read instructions or leaflets from hospitals or clinics	0.92	0.92	Excellent
	5	…have problems learning about your medical condition because of difficulty understanding health‐related written information	0.96	0.96	Excellent
IHL	6	*How easy would you say it is to* ^b^: …find related information when you have questions on disease or health problems	0.96	0.96	Excellent
	7	…find related information when you are not ill but want to do something to further improve your health	0.96	0.96	Excellent
	8	…give all the information a doctor, nurse or pharmacist need when you talk to them	0.88	0.87	Excellent
	9	…ask the questions you want to ask when you talk to a doctor, nurse or pharmacist	0.96	0.96	Excellent
	10	…extract the information you want when you talk to a doctor, nurse or pharmacist	0.96	0.96	Excellent
	11	…ask a doctor, nurse or pharmacist to further explain anything that you do not understand after talking with them	0.92	0.92	Excellent
	12	…understand the obtained information when you talk to a doctor, nurse or pharmacist	1.00	1.00	Excellent
CHL‐1	13	*When you get information for health in daily life, how often do you consider the following* ^c^: …whether the information source is credible	0.96	0.96	Excellent
	14	…whether the information content is valid and reliable	0.83	0.83	Excellent
	15	…whether the publish time is appropriate	0.79	0.79	Excellent
	16	…whether other reliable sources support the facts or conclusions of this source	0.88	0.87	Excellent
	17	…whether the person or organization that produced the information have a bias	0.83	0.83	Excellent
	18	…whether the information is applicable to you	0.83	0.83	Excellent
CHL‐2	19	*How do you agree about the following* ^d^: …socioeconomic status affects health	0.92	0.92	ExcellentExcellent
	20	…stress affects health	0.96	0.96	Excellent
	21	…being isolated from the community and workplace impacts health	0.92	0.92	Excellent
	22	…having little control over one's work impacts health	0.92	0.92	Excellent
	23	…poor childhood experience has an impact on one's physical/mental health when he or she becomes an adult	0.92	0.92	Excellent
	24	…good social relations contribute to health	0.96	0.96	Excellent
	25	…transportations impacts health	0.96	0.96	Excellent
CHL‐3	26	*How often do you* ^e^: …participate in government's programmes about health promotion and disease prevention	0.83	0.83	Excellent
	27	…participate in community's initiatives in health promotion and disease prevention	0.96	0.96	Excellent
	28	…participate in nongovernmental organizations' initiatives in health promotion and disease prevention	0.88	0.87	Excellent
	29	…help your family members or a friend when they had questions concerning health issues	0.96	0.96	Excellent
	30	…seek information from others when you come up with questions concerning a health issue	0.92	0.92	Excellent
	31	…share and communicate your opinion about illness when you talk to a family member or friend	0.92	0.92	Excellent

*Note*: a, response options range from ‘1 = always’ to ‘5 = never’; b, response options range from ‘1 = very difficult’ to ‘5 = very easy’; c, response options range from ‘1 = never’ to ‘5 = always’; d, response options range from ‘1 = strongly disagree’ to ‘5 = strongly agree’; d, response options range from ‘1 = never’ to ‘5 = always’.

Abbreviations: FHL, functional health literacy; IHL, interactive health literacy.

#### 3.2.1 Round 1

In Round 1, all 34 items were content‐validated (I‐CVI: 0.75–0.96; K* = 0.74–1.00) (see Table 2) based on the responses of all participants. Only one draft item on ‘Whether think about the information is valid’ possessed low content validity (I‐CVI < 0.79, K* = 0.74). This may have been caused by the difficulty in differentiating it from another draft item on ‘Whether think about the information is reliable’ in Chinese, as the majority of experts and laypeople highlighted. Thus, we combined these two items into one item (i.e., No. 14) to become ‘Whether think about the information is valid and reliable’. In addition, several items (i.e., No. 3, 4, 11, 15) were revised or rephrased since the panel members remarked that their wording remained vague or inappropriate in the text box. For instance, for one item in FHL (i.e., No. 3), one professor in Group A commented: ‘The scenario mentioned was not suitable in the local context. Citizens often need to read these instructions or leaflets from hospital and clinic, instead of pharmacy’. Thus, we changed ‘pharmacy’ to ‘clinic’ for the item. We added several examples to make certain items (i.e., No. 2, 5.8, 9, 23, 25) more specific as suggested by participants. Finally, a total of 33 items from 34 items were retained after Round 1.

#### 3.2.2 Round 2

The 33 items were rerated in Round 2 and content validities improved (I‐CVI: 0.79–1.00; K*: 0.74–1.00). In terms of the clarity on items, we mainly received positive comments. However, three items ‘How do you agree about the lesser the income the greater the tendency to become ill’, ‘How do you agree about socially vulnerable groups more likely turn to alcohol, drugs, and tobacco to relieve the pain of harsh economic and social conditions’ and ‘How do you agree about socially vulnerable groups more likely have no good eating habits and inadequate food supply to promote health and well‐being’ were criticized because of the overlapping and different interpretations of ‘socially vulnerable groups’. Therefore, we combined the three items into one item, ‘How do you agree about socioeconomic status affects health’, to make the item content more precise. Thus, the HLS‐HK included a total of 31 items from 33 items after Round 2.

#### 3.2.3 Round 3

In Round 3, each participant was asked to confirm the relevance of those items without changes and rerate the relevance with regard to the newly combined item resulting from Round 2. Eventually, for each item, over 70% of all participants agreed that it was relevant in Round 3. Thus, consensus was achieved for individual items and coverage. All 31 times showed excellent content validity (I‐CVI: 0.79–1.00; K*: 0.74–1.00) (see Table 2). We did not receive any further comments for adding or removing or revising items during this round. Thus, the Delphi exercise concluded with three rounds.

## 4 DISCUSSION

A validated and theoretically based HL scale, HLS‐HK was developed through a rigorous and systematic deductive approach and a modified e‐Delphi survey.

### 4.1 Bridging measurement gap

In comparison with the scales^30^, ^31^, ^32^, ^33^, ^34^, ^35^ based on Nutbeam's framework, the HLS‐HK fully operationalized the three content areas (i.e., FHL, IHL and CHL) in this framework. In the domain of FHL, we formulated five items to examine individuals' skills to read information, fill out forms and understand health‐related materials in healthcare settings. To measure the level of IHL, seven items were built to examine individuals' competencies to search for health‐related information and effectively communicate with healthcare workers.

More importantly, this scale bridged the measurement gap in the domain of CHL by providing the following multilevel subdomains. In the subdomain of CHL‐1, instead of simply asking the frequency to assess the trustworthiness of information like previous scales,^30^, ^35^ we generated seven items to assess subjects' behaviours to critically appraise information in terms of its resources, contents, publication date and publisher. Regarding the CHL‐2, as mentioned earlier, the knowledge of how social structural factors affect health was rarely thoroughly measured in HL measurement tools. By learning from one Japanese HL scale,^48^ we formulated seven items to directly test participants' knowledge about the impact of several significant social determinants of health. With respect to CHL‐3, we found that most of the current HL scales^30^, ^74^ only considered an individual's ‘collective action for health’ (i.e., collective efforts to create and preserve public goods, such as a clean environment and herd immunity) as the component of CHL. This might be because the current measurements were mainly developed in Western countries (e.g., the United States and Australia), where people are more open to social action or democratic participation. In this case, only focusing on ‘collective action for health’ cannot fully capture the CHL‐3 level of some population groups who have low interest in social movements or limited resources to participate, such as Hong Kong. Thus, we generated three new items (i.e., No. 29–31) to address social determinants of health at the interpersonal level (i.e., creating a supportive social network for health). In fact, the importance of abilities informing interpersonal level actions to address social determinants was addressed in one CHL scale targeting adolescents in Norway.^75^ However, those abilities were measured through items related to positive self‐beliefs to cope with a variety of situations to promote health in their social network and communities (e.g., ‘I am a person that can share information on factors that influence health with others’), rather than the real actions as our scale has done. However, we did encounter the challenges to thoroughly measuring CHL, which we have discussed below.

### 4.2 Including opinions of healthcare providers and consumers

Compared with the traditional Delphi method of only recruiting experts into the panel, we included healthcare professionals and the general population, who both play crucial roles in health‐related research. To achieve a representative sample, we recruited healthcare providers with diverse professional disciplines and laypeople with a balanced distribution of age and gender. In the progress, we made use of the opinions of all the agents involved and considered them all to be equal in the three‐round procedure. The two groups, however, did share different points of view on certain items which may be influenced by their professional or personal experience. Moreover, these differences are mainly reflected in CHL. For example, laymen representatives and healthcare professionals disagreed on item No. 18 (i.e., ‘Whether think about the information is applicable to you’). Laypeople mentioned that they usually randomly read the information during their daily life and did not think it is necessary to assess its applicability. By contrast, most experts commented that people should contextualize information for their own good and take actions after fully appraising the information in their own world. This disagreement might be explained by previous studies' findings, that is, even though people might know the strategies to check the quality of information, they do not routinely use these.^76^, ^77^ Hence, it is a question of how the scholarly discourse on information appraisal informs people's daily practice and reflects their relevant abilities. Another example is item No. 19 (i.e., ‘How do you agree with socioeconomic affects health’). Laypeople acknowledged the impact of socioeconomic factors but tend to feel that individuals' behaviours have a greater impact on health, while experts can thoroughly understand the influence of socioeconomic factors by analysing them from the perspective of health inequities. With relation to this point, Chinn^78^ suggested that asking about people's awareness of social determinants of health is methodologically tricky. Individuals who might struggle to link social disadvantage and health, are perhaps more likely to express such ideas through a contextualized narrative description of their own life experience instead of completing a fixed‐choice question.^78^ However, a narrative interview is a time‐consuming procedure that may not be applicable in a busy clinical setting. The above arguments about CHL indicated the complexities in operationalizing of this domain in a real‐world setting. We hope our work contributes to further exploring this operationalization from the laypeople and scholars' conceptions.

### 4.3 Implications

Based on the detailed literature review and our rigorous deductive approach, we extended Nutbeams' conceptual framework with 31 items. In the item generation process, we asked stakeholders' opinions to make sure our scale is content‐validated and user‐friendly. This is critical to build a native measurement and support local researchers, policymakers and practitioners to use this scale for relevant studies and health programmes. These items will undergo further rigorous testing in our target population groups in future studies. Other researchers can use or amend our scale for their research interests and validate the items in various settings and populations. It is thus reasonable to assume that our work can contribute to the further refinement of this conceptual model.

### 4.4 Limitations

Study limitations include the following: First, although we asked experts and laypeople to suggest additional items in the three‐round survey, no new items were added by them. This might be insufficient to create a tool that captures all skills related to HL. To enhance the comprehensiveness of a new tool, inductive methods (e.g., in‐depth interviews and focus groups) could be used in *Phase I*. Second, the decision to use an agreement index threshold of 0.70 used in this study was arbitrary. Owing to the diversity of topics covered by the Delphi method, there is no standard threshold for determining consensus.^79^ This study chose an acceptable threshold, as has been carried out in most studies.^55^, ^80^, ^81^, ^82^ Third, the panel members could not directly discuss any concerns or exchange opinions with other panellists because we conducted the Delphi study online. Although we provided feedback at the conclusion of each round, a structured meeting after the first two rounds may facilitate deeper discussions among the panel members. Fourth, selection biases might exist in the Delphi panellists because we conducted a nonprobability sampling technique. For example, although we intended to achieve a balance of education attainment in Group B, the actual proportion of the participants who were well‐educated was high because sufficient reading levels and cognitive skills were needed to judge the reference of each item. To make sure the scale is suitable to use in the entire population, we will examine its psychometric proprieties among the general population using quota sampling. Additionally, we only included healthcare scholars and clinical workers in Group A because of limited resources. To achieve a deeper understanding of the skills that people need to find and use health‐related information in various settings, future studies should consider including a broader range of healthcare providers (e.g., allied health) in Group A. Fifth, the lack of item deduction in this Delphi process highlights the need for future studies such as cognitive interviews and psychometric properties testing to achieve further item reduction.

## 5 CONCLUSION

By combining a literature review and a Delphi survey, this study identified a set of content validity items for the HLS‐HK. Specifically, the review ensured that all draft items were generated based on scientific evidence. The mixed method approach using a three‐round survey provided quantitative and qualitative data which led to item modification and improved content validity. Compared with previous HL scales, this newly developed scale fully operationalized the skills involved in FHL, IHL and CHL. It is useful to examine people's HL levels and identify the barriers that they may encounter in processing health‐related information to make appropriate health‐related decisions. The next steps in the research will involve testing its face validity for respondents, and psychometric properties to identify its final version and more parsimonious form.

## AUTHOR CONTRIBUTIONS

Eliza Lai‐Yi Wong, Phoenix K.‐H. Mo, Dong Dong and Cindy Yue Tian designed the Delphi study and acquired funding. Cindy Yue Tian performed the literature review and three‐round survey, analysed and synthesized the data and wrote the draft manuscript. Eliza Lai‐Yi Wong was responsible for data analysis, data curation and project administration. Dong Dong and Phoenix K.‐H. Mo were in charge of project administration and supervision. Richard H. Xu provided guidance on the three‐round surveys. Annie Wai‐Ling Cheung assisted with the funding acquisition and project administration. Eliza Lai‐Yi Wong, Phoenix K.‐H. Mo, Dong Dong, Richard H. Xu and Annie Wai‐Ling Cheung commented and edited the whole draft. Cindy Yue Tian critically revised the manuscript. All authors read and approved the final manuscript.

## CONFLICT OF INTEREST

The authors declare no conflict of interest.

## ETHICS STATEMENT

This study was conducted in accordance with the Declaration of Helsinki and approved by the Survey and Behavioral Research Ethics Committee of the Chinese University of Hong Kong (Reference No. SBRE‐20‐793). Informed consent for the study participation was obtained before the survey.

## Supporting information

Supporting information.

Supporting information.

Supporting information.

## Data Availability

The data sets generated and/or analysed during the current study are not publicly available to protect the anonymity of participants but are available from the corresponding author on reasonable request.
