# Supplementary material for: Developing a Health Literacy Scale for adults in Hong Kong: A modified e‐Delphi study with healthcare consumers and providers
Source: Health Expect. 2022 Nov 8;26(1):245–55. doi: 10.1111/hex.13651 (PMC9854330; doi:10.1111/hex.13651)
Supplement: Supplementary file 1 — Supporting information. [file HEX-26--s001.docx]

**Appendix 1. Results of the content of items: deductive approach in Phase I**

| **Domain** | **Sample** | **Coding** | **Grouping** | **Results** | |
| --- | --- | --- | --- | --- | --- |
|  |  |  |  | Content categories | Description |
| FHL | AAHLS ^1^ | "need some to help to read" | “need help to read” | "need help to read"; "need help to fill in documents"; "find characters that cannot to read"; "find content is too difficult to understand"; "difficulty in understanding written information" | Abilities to read health-related information;  to fill out medical forms; to read characters and understand the content in instructions or leaflets from hospital or pharmacy; to understand the written information in health-related documents. |
|  |  | "need help to fill in official documents" | "need help to fill in documents" |  |  |
|  | HLS-14 ^2^ | "find characters that cannot to read" | "find characters that cannot to read" |  |  |
|  |  | "find the print is too small to read" | "need help to read" |  |  |
|  |  | "find the content is too difficult to understand" | "find content is too difficult to understand" |  |  |
|  |  | "takes a long time to read" | "difficulty in understanding written information" |  |  |
|  |  | "need someone to help to read" | "need help to read" |  |  |
|  | c-HLAT-8 ^3^ | "understand written information that comes with medication" | "difficulty in understanding written information" |  |  |
|  |  | "understand information presented in health pamphlets" | "difficulty in understanding written information" |  |  |
|  | BHLQ ^4^ | "need someone to help to read" | “need help to read” |  |  |
|  |  | "difficulty in understanding written information" | "difficulty in understanding written information" |  |  |
| IHL | AAHLS ^1^ | "give information" | "give information" | "find information"; "give information"; "ask questions"; "ask for explanation"; "extract information"; "understand information" | Abilities to find health-related information; to provide information, ask questions, seek explanations, extract useful information, and understand the obtained information when talking with healthcare workers. |
|  |  | "ask questions" | "ask questions" |  |  |
|  |  | "ask for explanation" | "ask for explanation" |  |  |
|  | HLS-14 ^2^ | "collect information" | "find information" |  |  |
|  |  | "extract information" | "extract information" |  |  |
|  |  | "understand information" | “understand information" |  |  |
|  |  | "communicate opinions" | "give information" |  |  |
|  | c-HLAT-8 ^3^ | "find information" | "find information" |  |  |
| CHL-1 | AAHLS ^1^ | "make sense in your situation" | "applicable" | "credible"; "reliable";  "valid"; "applicable";  “biased”  “sources”  “publish date”  “content” | Abilities to examine the quality of the information: whether the source is credible; whether the content is reliable and valid; whether the publish date is valid; whether the people or organization who published the information is biased. |
|  |  | "can be trusted" | "credible" |  |  |
|  | HLS-14 ^2^ | "applicable" | "applicable" |  |  |
|  |  | "credible" | "credible" |  |  |
|  |  | "valid" | "valid" |  |  |
|  |  | "reliable" | "reliable" |  |  |
|  | c-HLAT-8 ^3^ | "compare the data from various sources" | "sources" |  |  |
|  | CRAAP ^5^ | "timeliness of the information" | "publish date" |  |  |
|  |  | "relate to your topic or answer your question" | "applicable" |  |  |
|  |  | "source of the information" | "sources" |  |  |
|  |  | "reliability, truthfulness and correctness of the content" | "content" |  |  |
|  |  | “biased” | “biased” |  |  |
| CHL-2 | HL-SDHQ ^6^ | "social gradient" | "social gradient" | "social gradient"; "stress"; "early life"; "social exclusion"; "work"; "social support"; "addition"; "food"; "transport". | Knowledge about the impact of “social gradient”, “stress”, “early life”, “social exclusion”, “work”, “social support”, “addition”, “food”, and “transport” on health. |
|  |  | "stress" | "stress" |  |  |
|  |  | "early life" | "early life" |  |  |
|  |  | "social exclusion" | "social exclusion" |  |  |
|  |  | "work" | "work" |  |  |
|  |  | "social support" | "social support" |  |  |
|  |  | "addition" | "addition" |  |  |
|  |  | "food" | "food" |  |  |
|  |  | "transport" | "transport" |  |  |
| CHL-3 | IHLQ ^7^ | "participation in public festivals" | “action for health at the social level" | "action for health at the social level"; “action for health at the interpersonal level". | Abilities to participate in activities to address social determinants of health at the social level and the interpersonal level. |
|  |  | "participation in the meetings of local health centers" | “action for health at the social level" |  |  |
|  | c-HLAT-8 ^3^ | "help family members or friend if they had questions concerning health issues" | “action for health at the interpersonal level" |  |  |
|  |  | "get information and advice from others for your health issues" | “action for health at the interpersonal level" |  |  |

Reference:

[1] Chinn D, McCarthy C. All Aspects of Health Literacy Scale (AAHLS): developing a tool to measure functional, communicative and critical health literacy in primary healthcare settings. Research Support, Non-U.S. Gov't. Patient Education & Counseling. 2013;90(2):247-53.

[2] Suka M, Odajima T, Kasai M, et al. The 14-item health literacy scale for Japanese adults (HLS-14). Article. *Environmental Health and Preventive Medicine*. 2013;18(5):407-415. doi:10.1007/s12199-013-0340-z

[3] Abel T, Hofmann K, Ackermann S, Bucher S, Sakarya S. Health literacy among young adults: a short survey tool for public health and health promotion research. Health Promotion International. 2015;30(3):725-735.

[4] Chew LD, Bradley KA, Boyko EJ. Brief questions to identify patients with inadequate health literacy. health. 2004;11:12.

[5] Blakeslee S. The CRAAP test. Loex Quarterly. 2004;31(3):4.

[6] Matsumoto M, Nakayama K. Development of the health literacy on social determinants of health questionnaire in Japanese adults. BMC Public Health. 2017;17(1):30.

[7] Haghdoost AA, Rakhshani F, Aarabi M, et al. Iranian health literacy questionnaire (IHLQ): An instrument for measuring health literacy in Iran. Iranian Red Crescent Medical Journal. 2015;17(6).
