# Supplementary material for: Developing a Health Literacy Scale for adults in Hong Kong: A modified e‐Delphi study with healthcare consumers and providers
Source: Health Expect. 2022 Nov 8;26(1):245–55. doi: 10.1111/hex.13651 (PMC9854330; doi:10.1111/hex.13651)
Supplement: Supplementary file 2 — Supporting information. [file HEX-26--s002.docx]

**Appendix 2 Wording changes of HLS-HK (three-round survey)**

| Domain | No. | Round 1 | Round 2 | Round 3 |
| --- | --- | --- | --- | --- |
| FHL | 1 | How often do you ^a^:  …need help when you are given information to read by your doctor, nurse or pharmacist | Added example of “help” : e.g., asking for an explanation of the contents of the information | No change |
|  | 2 | …need help when you are asked to fill out medical forms by your doctor, nurse or pharmacist | Added example of “help” : e.g., asking for an explanation of the contents of the medical forms | No change |
|  | 3 | …find that characters cannot understand when you read instructions or leaflets from hospitals or pharmacies | "pharmacies" changed to "clinics" ^[1]^ | No change |
|  | 4 | …feel that the content is too difficult to understand when you read instructions or leaflets from hospitals or pharmacies | "pharmacies" changed to "clinics" ^[2]^ | No change |
|  | 5 | … have problems learning about your medical condition because of difficulty understanding health-related written information | Added example of “health-related written information” (e.g., medical report) | No change |
| IHL | 6 | How easy would you say it is to ^b^:  …find related information when you have questions on disease or health problems | No change | No change |
|  | 7 | …find related information when you are not ill but want to do something to further improve your health | No change | No change |
|  | 8 | …give all the information a doctor, nurse, or pharmacist need when you talk to them | Added examples of “information” : e.g., why you were ill and what medicine you had taken | No change |
|  | 9 | …ask the questions you want to ask when you talk to a doctor, nurse, or pharmacist | Added examples of “questions” : e.g., the causes of disease, the side effects of medicine | No change |
|  | 10 | …extract the information you want when you talk to a doctor, nurse, or pharmacist | No change | No change |
|  | 11 | … ask a doctor, nurse, or pharmacist explain anything that you do not understand | Changed to "…ask a doctor, nurse, or pharmacist to further explain anything that you do not understand after talking with them" ^[3]^. | No change |
|  | 12 | …understand the obtained information when you talk to a doctor, nurse, or pharmacist | No change | No change |

**Appendix 2 (continued)**

| Domain | No. | Round 1 | Round 2 | Round 3 |
| --- | --- | --- | --- | --- |
| CHL-1 | 13 | When you get information for health in daily life, how often do you consider the following ^c^:  …whether the information source is credible | No change | No change |
|  | 14 | … whether the information content is valid | Combined the two items as "whether the information content is valid and reliable" ^[4]^ | No change |
|  |  | … whether the information content is reliable |  |  |
|  | 15 | … whether the publish time is valid | "valid" changed to "appropriate" ^[5]^ | No change |
|  | 16 | … whether other sources support the facts or conclusions of this source | No change | No change |
|  | 17 | …whether the person or organization that produced the information have a bias | No change | No change |
|  | 18 | …whether the information is applicable to you | No change | No change |
| CHL-2 | 19 | How do you agree about the following ^d^:  … the lesser the income the greater the tendency to become ill | No change | Combined the three items as "socioeconomic status (e.g., educational attainment, income) affects health" ^[6]^ |
|  |  | … socially vulnerable groups more likely turn to alcohol, drugs, and tobacco to relieve the pain of harsh economic and social conditions |  |  |
|  |  | .. socially vulnerable groups more likely have no good eating habits and inadequate food supply to promote health and well-being |  |  |
|  | 20 | …stress affects health |  |  |
|  | 21 | …being isolated from the community and workplace impacts health | No change | No change |
|  | 22 | …having little control over one’s work impacts health | No change | No change |
|  | 23 | …poor childhood experience has an impact on one's physical/mental health when he or she becomes an adult | Added examples of “experience” : e.g., nutritional deficiency , emotional deprivation | No change |
|  | 24 | …good social relations contribute to health | No change | No change |

**Appendix 2 (continued)**

| Domain | No. | Round 1 | Round 2 | Round 3 |
| --- | --- | --- | --- | --- |
| CHL-2 | 25 | …transportations impacts health | Added examples of “transportation” : e.g., circling and walking | No change |
| CHL-3 | 26 | How often do you ^e^:  …participate in government’s programmes about health promotion and disease prevention | No change | No change |
|  | 27 | …participate in community’s initiatives in health promotion and disease prevention | No change | No change |
|  | 28 | …participate in non-governmental organisations’ initiatives in health promotion and disease prevention | No change | No change |
|  | 29 | …help your family members or a friend when they had questions concerning health issues | No change | No change |
|  | 30 | …seek information from others when you come up with questions concerning a health issue | No change | No change |
|  | 31 | …share and communicate your opinion about illness when you talk to a family member or friend | No change | No change |

Notes:

a = response options range from “1 = always” to “5 = never”; b = response options range from “1 = very difficult” to “5 = very easy”; c = response options range from “1 = never” to “5 = always”; d = response options range from “1 = strongly disagree” to “5 = strongly agree”; d = response options range from “1 = never” to “5 = always”.

[1] = Reason: One expert in Group A commented: “The scenario mentioned was not suitable in the local context. Citizens often need to read these instructions or leaflets from hospital and clinic, instead of pharmacy”.

[2] = Reason: One expert in Group A commented: “The scenario mentioned was not suitable in the local context. Citizens often need to read these instructions or leaflets from hospital and clinic, instead of pharmacy”.

[3] = Reason: One expert in Group A asked, “Does asking for an explanation for what you don't understand also fall within the scope of asking a question?”. Actually, the item about seeking explanation was designed as a follow-up question to the item about asking questions by testing the ability to reflect on healthcare professionals’ answers.

[4] = Reason: The majority of experts and laypeople highlighted that it was difficult to differentiate the two items in Chinese.

[5] = Reason: Most of the participants commented that the translation of “valid” was not culturally adapted. To avoid ambiguity of the translation in the local context, we used “appropriate” to replace “valid”.

[6] = Reason: The three items were criticized because of the overlapping and different interpretations of “socially vulnerable groups”.
