# Supplementary material for: Developing a Health Literacy Scale for adults in Hong Kong: A modified e‐Delphi study with healthcare consumers and providers
Source: Health Expect. 2022 Nov 8;26(1):245–55. doi: 10.1111/hex.13651 (PMC9854330; doi:10.1111/hex.13651)
Supplement: Supplementary file 3 — Supporting information. [file HEX-26--s003.docx]

# **Health Literacy** **Scale-Hong Kong 香港居民健康素養量表**

How often do you 以下情況有幾經常在您的生活中出現？

|  | **Always**  **總是** | **Often**  **經常** | **Sometimes**  **有時** | **Seldom**  **很少** | **Never**  **從不** |
| --- | --- | --- | --- | --- | --- |
| 1. … need someone to help when you are given information to read by doctor, nurse, or pharmacist (e.g., asking for an explanation of the contents of the information) 當醫生、護士或藥劑師請您閱讀醫療資訊時, 您需要協助（例如對資訊作出解釋) |  |  |  |  |  |
| 1. … need someone to help when you are asked to fill out medical forms by doctor, nurse, or pharmacist (e.g., asking for an explanation of the contents of the medical forms) 當醫生、護士或藥劑師請您填寫醫療表格時, 您需要協助(例如對表格填寫內容作出解釋) |  |  |  |  |  |
| 1. … find that characters cannot read when you read instructions or leaflets from hospitals or clinics當閱讀醫院或診所的指引或宣傳單張時，您發現有不認識或不理解的字詞 |  |  |  |  |  |
| 1. … feel that the content is too difficult to understand when you read instructions or leaflets from hospitals or clinics當閱讀醫院或診所的指引或宣傳單張時，您覺得內容難以理解 |  |  |  |  |  |
| 1. … have problems learning about your medical condition because of difficulty understanding health-related written information (e.g., medical report) 由於理解醫療相關的書面資料(例如醫療報告)有困難，您無法瞭解您的健康狀況 |  |  |  |  |  |

How easy would you say it is to 以下情況在您的日常生活中有幾容易完成？

|  | **Very difficult**  **非常難** | **Difficult**  **有點困難** | **Neutral**  **中立** | **Easy**  **還算容易** | **Very**  **easy**  **非常容易** |
| --- | --- | --- | --- | --- | --- |
| 1. … find related information when you have questions on disease or health problems當您對疾病或健康問題有疑問時，找到相關的資訊 |  |  |  |  |  |
| 1. … find related information when you are not ill but want to do something to further improve your health 當您沒有生病但想進一步改善您的健康時，找到相關的資訊 |  |  |  |  |  |
| 1. … give a doctor, nurse, or pharmacist all the information they need (e.g., why you were ill and what medicine you had taken) 向醫生、護士或藥劑師提供所需的資訊(例如您哪裡不舒服，吃了什麼藥) |  |  |  |  |  |
| 1. … ask a doctor, nurse, or pharmacist the questions you want to ask (e.g., the causes of disease, the side effects of medicine) 向醫生、護士或藥劑師表達您想問的問題(例如造成疾病的原因，藥物副作用) |  |  |  |  |  |
| 1. ... extract the information you want when you talk to a doctor, nurse, or pharmacist 和醫生、護士或藥劑師溝通時，提取您需要的資訊 |  |  |  |  |  |
| 1. … ask a doctor, nurse, or pharmacist to further explain anything that you do not understand after talking with them主動讓醫生、護士或藥劑師對您不理解的資訊作出解釋 |  |  |  |  |  |
| 1. … understand the obtained information when you talk to a doctor, nurse, or pharmacist理解醫生、護士或藥劑師和您溝通時提供的資訊 |  |  |  |  |  |

When you get information for health in daily life, how often do you consider the following當您獲得有關健康的資訊時，您有幾經常考慮以下情況？

|  | **Never**  **從不** | | **Seldom**  **很少** | | | **Sometimes**  **有時** | **Often**  **經常** | **Always**  **總是** |
| --- | --- | --- | --- | --- | --- | --- | --- | --- |
| 1. … whether the information source is credible資訊來源是否可信任 | |  | |  |  | |  |  |
| 1. … whether the information content is valid and reliable 資訊內容是否有效可靠 | |  | |  |  | |  |  |
| 1. … whether the publish time is appropriate資訊發布時間時間是否適合 | |  | |  |  | |  |  |
| 1. … whether other sources support the facts and/or conclusions of this source是否有其他資訊來源支持所獲取的資訊 | |  | |  |  | |  |  |
| 1. … whether the person or organization that produced the information have a bias發佈信息的個人或團體會否偏頗 | |  | |  |  | |  |  |
| 1. … whether the information is applicable to you資訊對您個人是否適用 | |  | |  |  | |  |  |

How do you agree or disagree about the following 對以下說法您有幾大程度同意？

|  | **Totally disagree**  **完全**  **不同意** | **Disagree**  **不同意** | **Neutral**  **中立** | **Agree**  **同意** | **Totally agree**  **完全**  **同意** |
| --- | --- | --- | --- | --- | --- |
| 1. … socioeconomic status （e.g., educational attainment, income） affects health社會經濟地位(例如教育程度、收入水平) 會影響健康 |  |  |  |  |  |
| 1. … stress affects health壓力會影響健康 |  |  |  |  |  |
| 1. … being isolated from the community and workplace impacts health於社區和工作場所被孤立會對健康產生影響 |  |  |  |  |  |
| 1. … having little control over one’s work causes depression and impacts health對工作有無力感會影響健康 |  |  |  |  |  |
| 1. …poor childhood experience (e.g., nutritional deficiency , emotional deprivation) has an impact on one's physical/mental health when he or she becomes an adult不良的童年經歷(例如營養缺乏、缺乏關愛)會影響成年身體/精神健康 |  |  |  |  |  |
| 1. … good social relations contribute to health良好的社交關係有益於健康 |  |  |  |  |  |
| 1. … transportation (e.g., circling and walking) impacts health出行方式(例如踩單車、步行) 會影響健康 |  |  |  |  |  |

How often do you 以下情況有幾經常在您的生活中出現？

|  | **Never**  **從不** | **Seldom**  **很少** | **Sometimes**  **有時** | **Often**  **經常** | **Always**  **總是** |
| --- | --- | --- | --- | --- | --- |
| 1. … participate in government’s programmes in health promotion and disease prevention參與促進政府制定有利健康的公共政策的活動 |  |  |  |  |  |
| 1. … participate in your community’s initiatives in health promotion and disease prevention參與社區的有關促進健康和疾病預防的活動 |  |  |  |  |  |
| 1. … participate in non-governmental organisations’ activities in health promotion and disease prevention參與非牟利的及非政府機構的有關健康和疾病預防相關的活動 |  |  |  |  |  |
| 1. … help your family members or a friend when they had questions concerning health issues您的家人或朋友對健康問題有疑問時，您會幫助他們 |  |  |  |  |  |
| 1. … seek information from others when you come up with questions concerning a health issue您對健康問題有疑問，您會向周圍的人尋求相關 |  |  |  |  |  |
| 1. … share and communicate your opinion about illness when you talk to a family member or friend您與家人、朋友溝通時，您會與他們分享及交流您對疾病的看法 |  |  |  |  |  |
